# Comprehensive Clinical, Diagnostic, and In Silico Assessment of a Novel 1p36.33p36.32 Copy Number Variant

**DOI:** 10.1111/jcmm.71079

**Published:** 2026-02-25

**Authors:** Atieh Eslahi, Mir Salar Kahaei, Bita Barazandeh Shirvan, Masoome Alerasool, Sepideh Tabarestani, Razie Rezaie, Narges Hashemi, Javad Akhondian, Mobina Arabi, Farnoosh Ebrahimzadeh, Mehran Beiraghi Toosi, Majid Mojarrad

**Affiliations:** ^1^ Department of Medical Genetics, Faculty of Medicine Mashhad University of Medical Sciences Mashhad Iran; ^2^ Rare Pediatric Neurological Diseases Research Center Mashhad University of Medical Sciences Mashhad Iran; ^3^ Genetic Foundation of Khorasan Razavi Mashhad Iran; ^4^ Department of Pediatrics Dr Sheikh Hospital, Mashhad University of Medical Sciences Mashhad Iran; ^5^ Neuroscience Research Centre Mashhad University of Medical Sciences Mashhad Iran; ^6^ Blood Transfusion Organization Mashhad Iran; ^7^ Department of Pediatrics, Faculty of Medicine Mashhad University of Medical Sciences Mashhad Iran; ^8^ Department of Internal Medicine, Faculty of Medicine Mashhad University of Medical Sciences Mashhad Iran

**Keywords:** 1p36.33 duplication, array CGH, developmental delay, facial dysmorphism

## Abstract

Clinical manifestations of 1p36.33 duplications vary depending on duplication size. This region is prone to copy number variants associated with diverse phenotypes. We report a novel 1p36.33p36.32 duplication in a patient with developmental delay and facial dysmorphism. The causative duplication was detected by whole‐genome Oligo‐array CGH and confirmed by real‐time PCR. Integrative bioinformatic analyses—including network analysis, phenotype‐driven gene prioritisation, and dosage sensitivity assessment—were performed to explore the molecular basis of the phenotype; we used integrative bioinformatic analyses, including network analysis, phenotype‐driven gene prioritisation, and dosage sensitivity assessment. Assessment of a child with tonic seizures, developmental delay, and dysmorphic facial traits revealed a 2.3 MB gain in the 1p36.33p36.32 region (nucleotide 898,721 to 3,153,945) through array CGH. Bioinformatic analyses identified several candidate genes, including *GABRD*, *DVL1*, and *GNB1*, which are implicated in neurodevelopmental and congenital disorders. Pathway enrichment analysis revealed significant involvement of the ‘1P36 Copy Number Variation Syndrome’ pathway. This case expands the phenotypic spectrum of 1p36 duplications and highlights the importance of integrating clinical, genomic, and bioinformatic data for accurate interpretation.

## 1 Introduction

The 1p36 chromosomal region is susceptible to copy number alterations, encompassing both duplications and deletions, as well as rearrangements. A prevalent genetic anomaly in this area, 1p36 microdeletion, is associated with intellectual disability and developmental delay in infants [1, 2]. Conversely, the less common 1p36 microduplication can result in severe psychomotor delay and speech issues [3, 4]. Copy number variations in this locus correlate with a spectrum of phenotypic features, including hypotonia, spastic paralysis, optic atrophy, cerebellar atrophy, peripheral neuropathy, and syndromic neurological disorders [5]. Additionally, this region harbours genes with diverse copy number variations, such as *GABRD, KCNAB2, PLCH2*, protein kinase C (*PKC)‐zeta*, *ATAD3*, and *SKI* (v‐ski sarcoma viral oncogene homologue), each contributing to distinct clinical manifestations [6, 7]. For instance, Protein Kinase C (PKC)‐zeta, located in 1p36.33, acts as an atypical member of the PKC family crucial for cell proliferation, survival, and immune response [8]. Therefore, the clinical symptoms associated with the duplication of 1p36.33 seem variable depending on the variation size and the genes involved. Advancements in diagnostic techniques, like array comparative genome hybridisation (array‐CGH), have surpassed traditional karyotyping, enabling the detection of microdeletions, microduplication, and precise breakpoint rearrangements [9]. Here, we highlight a case presenting with neurodevelopmental delay, broad thumbs, esotropia, and a club foot on the right side that had been corrected with surgery. The patient carries a novel heterozygote copy number variant in the 1p36.33 region, as identified by array CGH. Duplication has been confirmed using quantitative Real‐Time PCR confirming a duplication in the *PRKCZ* gene. To elucidate the potential functional consequences of this duplication, we performed a comprehensive bioinformatic analysis, including protein–protein interaction network construction (STRING, MCODE), phenotype‐driven gene prioritisation (PhenoLyzer), assessment of gene dosage sensitivity (DECIPHER), and pathway enrichment analysis (Enrichr). Additionally, to identify OMIM‐morbid genes within the duplicated region, we used genescout. This integrative approach aimed to identify the candidate genes and biological pathways contributing to the observed phenotype.

## 2 Methods

### 2.1 Subject and Blood Sampling

We present clinical features and molecular findings in a 2‐year, 10‐month‐old female with copy number variation in 1p36 that were not previously described in the literature. The patient's peripheral blood was collected into an EDTA tube for genomic DNA extraction using Simbiolab kit. The quantity of DNA in the sample was determined using Nanodrop spectrophotometer. Additionally, agarose gel electrophoresis was used for the assessment of DNA quality. Informed consent has been taken from the patient's parents, allowing to publishing of the patient's clinical and genetic data.

### 2.2 Copy Number Variation Analysis Using Array CGH

The SurePrint G3 ISCA V2 8X60K whole‐genome Oligo‐Array version 2 (Agilent Technologies, Santa Clara, CA) was employed for whole‐genome oligonucleotide‐based array comparative genomic hybridisation (oligo‐array CGH) analysis. This array comprises approximately 60,000 oligonucleotide probes with an overall median probe spacing of 60 kb across the genome to 500 targeted disease regions. Genomic DNA from the patient and a male reference sample were labelled with Cy5 and Cy3 dyes, respectively, using the Agilent SureTag DNA Labeling Kit (Agilent Technologies) according to the manufacturer's instructions. Following the manufacturer's protocol, the patient's DNA was hybridised against the male reference sample (9399). After hybridisation, the arrays were washed using Agilent wash buffers and scanned using the Agilent SureScan Microarray Scanner (Agilent Technologies) at a resolution of 3 μm. The resulting images were processed using Agilent Feature Extraction software (version 12.0.3.1) to obtain the raw data files.

The raw data files were analysed using Agilent CytoGenomics software (version 4.0.3.12) with the following settings: ADM‐2 algorithm, threshold of 6.0, and a minimum of 4 consecutive probes for called intervals. The log2 ratio of patient/reference signal intensities was calculated, and copy number variations (CNVs) were identified based on the log2 ratio values and their statistical significance.

### 2.3 Quantitative Real‐Time PCR Validation

To validate copy number changes in the 1p36 region, relative quantitative real‐time PCR (qPCR) assays were conducted using the *PRKCZ* (protein kinase C, zeta) gene as the target and the *KMT2D* as the reference gene. The primers were designed with Primer3 software (version 0.4.0) and their sequences were aligned against the entire human genome using the UCSC program (Table S1). The qPCR reactions were set up in 20 μL volumes containing SYBR Green PCR Master Mix (2×), 0.25 μM of each primer, nuclease‐free water, and DNA template (50 ng/reaction). The thermal cycling conditions included an initial denaturation at 95°C for 15 min, and then 35 cycles of 95°C for 30 s and 60°C for 30 s. The qPCR assays were performed using MicPCR equipment. In addition to the test samples, a negative control sample from a normal individual without abnormality in 1p36 was included.

### 2.4 In Silico Analysis

To systematically prioritise candidate genes within the duplication region identified by array‐CGH, we performed a multi‐step bioinformatics analysis:

#### 2.4.1 Protein–Protein Interaction (PPI) Network Analysis

To comprehensively characterise the duplicated region, we first identified all genes located within the interval using the Ensembl genome browser [10], focusing specifically on protein‐coding genes. Protein interaction networks for these genes were generated using the STRING database [11] and visualised in Cytoscape (v3.10.3) [12], with MCODE [13] applied to detect hub genes and densely connected modules, highlighting potential driver genes within the region.

#### 2.4.2 OMIM Morbid Gene Identification

Genescout [14] was used to systematically identify OMIM‐morbid genes within the duplicated region.

#### 2.4.3 Phenotype‐Driven Gene Prioritisation

To assess genotype–phenotype correlations, we utilised Phenolyzer [15] by providing patient‐specific phenotypes (HPO terms), allowing prioritisation of genes most likely associated with the clinical features observed (Table S2).

#### 2.4.4 Assessment of Gene Dosage Sensitivity and CNV Phenotype Annotation

The DECIPHER database [16] was queried to identify genes within the duplicated region that have documented triplo‐sensitivity or dosage sensitivity. In addition, all reported clinically relevant CNVs overlapping the duplicated region were filtered and systematically reviewed. Associated phenotypic features for each CNV were extracted and summarised in a comparative table.

#### 2.4.5 Pathway Enrichment Analysis

Enrichr [17] was used for pathway enrichment analysis of protein‐coding genes in the duplicated region, focusing on WikiPathways 2024 Human and other relevant pathways.

## 3 Results

### 3.1 Clinical Evaluation

**FIGURE 1:**
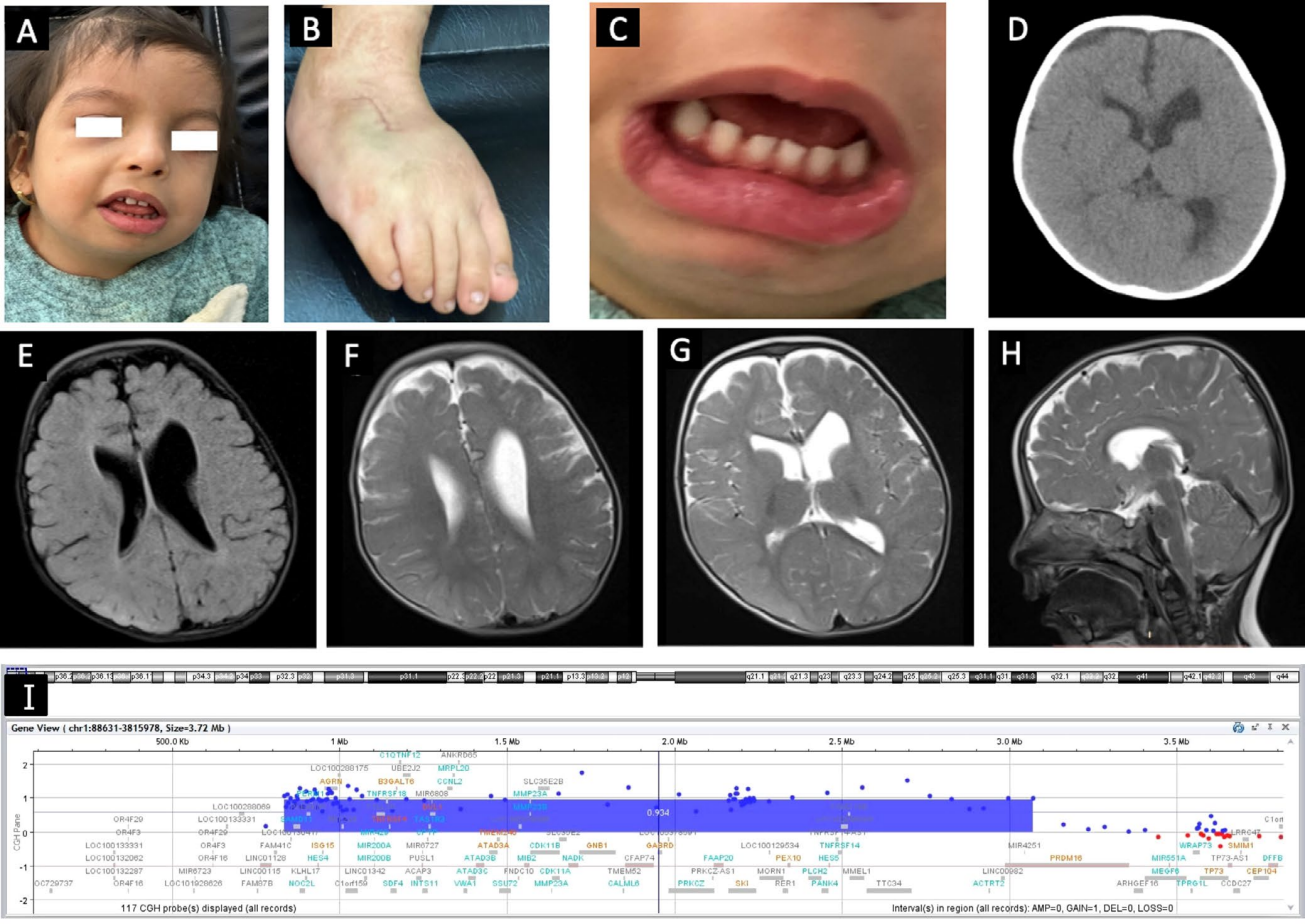
Clinical photographs, brain MRI and CT scan, and array‐CGH results of the patient with 1p36.33 duplication. Clinical photographs showing facial dysmorphism (A), surgically corrected right clubfoot (B), and widely spaced teeth (C). The axial brain CT scan shows left‐side ventriculomegaly without any calcification (D), and the T1 (E) and T2 (F, G, H) sequences of brain MRI show ventriculomegaly especially on the left side with a mild abnormality of cortical gyration on the frontal region. Array‐CGH results indicate a duplication at the 1p36.33p36.32 (GRCh37/hg19; chr1:834,101–3,070,509) (I); genomic coordinates elsewhere in the manuscript are reported on (GRCh38/hg38).

The patient, a 2‐year, 10‐month‐old female, was born in a non‐consanguineous family. The parents had two other healthy children, with no history of abortion. The patient exhibited distinctive features including micrognathia, pronounced lower lip, bulbous nose, ample cheeks, widely spaced teeth, a unilateral club foot affecting the right side, and esotropia (Figure 1A–C). At 6 months, she experienced her first tonic seizure that recurred at 2 years, 9 months. The patient's birth weight was 2700 g, last investigated weight was 10 kg, and head circumference was 44 cm. At the age of 2 years, 10 months, she could only crawl with poor social skills. Her phonetic and phonological abilities were limited to crying and babbling, although her social attention was relatively preserved. The cause of the neurodevelopmental delay and tonic seizures was determined by brain MRI and CT, EEG, echocardiogram, and laboratory testing. Laboratory tests revealed normal organic acid levels in urine, while a brain CT scan and MRI showed left ventriculomegaly with a mild abnormality of cortical gyration in the frontal region (Figure 1D–H). Echocardiography showed no abnormalities, and EEG indicated scattered nonspecific sharp waves.

### 3.2 CNV Analysis Using Array CGH and PCR Validation

Molecular cytogenetic investigation using Array‐CGH revealed a 2.3 Mb duplication CNV at the region 1:898,721–3,153,945 (2.3 Mb), localised in (1p36.33‐p36.32) (Figure 1I). According to ACMG/ClinGen CNV interpretation guidelines [18], the variant met criteria 1A, 2B, 3C, and 4L, leading to its classification as pathogenic. The duplication encompassed 63 protein‐coding genes and partially overlapped an established ClinGen triplosensitive genomic region (ISCA‐37434, 1p36 terminal region). Consistent with the CNV gain scoring metric, this overlap was captured as Evidence 2B (Table S3). Case–control data demonstrated enrichment among affected individuals with consistent phenotypes, and no overlap was detected with common population variants in gnomAD or DGV. Detailed ACMG/ClinGen scoring is provided in Table S3. Real‐time PCR results on the *PRKCZ* gene confirmed Array‐CGH results.

### 3.3 In Silico Approaches to Array‐Based Candidate Genes

#### 3.3.1 Network‐Based Identification of Key Genes in a Disease Relevant CNV Region

**FIGURE 2:**
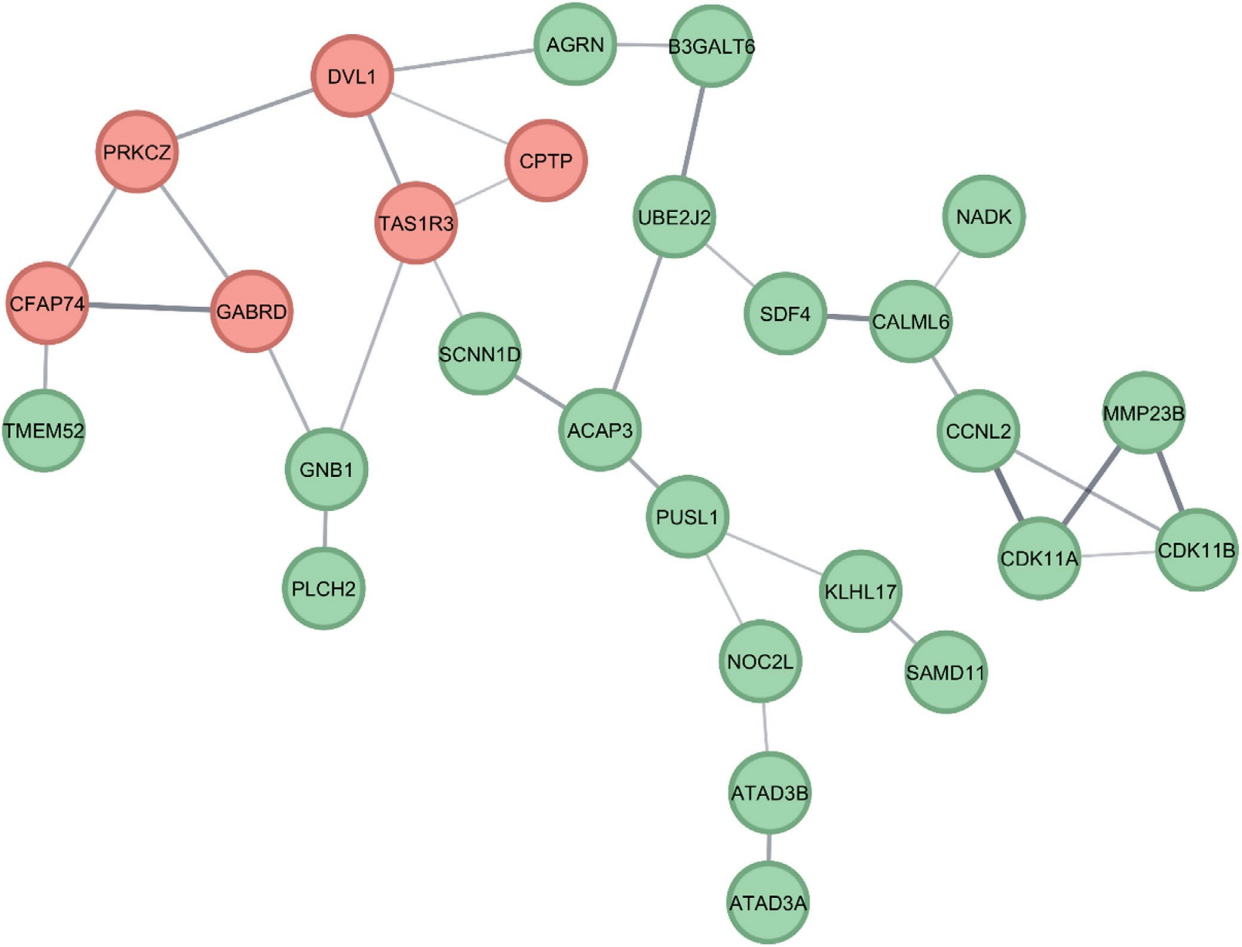
Protein–protein interaction (PPI) network of the 27 protein‐coding genes within the duplicated region, constructed using STRING and visualised in Cytoscape. Nodes represent genes; edges represent predicted interactions. Hub genes identified by MCODE are highlighted in red.

A total of 89 genes were identified within the duplicated region using Ensembl (hg38), of which 63 were protein‐coding. These 63 protein‐coding genes were subjected to protein–protein interaction (PPI) network analysis using the STRING database. The initial PPI network included several genes with no significant interactions; these disconnected nodes were excluded from further analysis. The final PPI network comprised 27 interconnected nodes and 32 edges, with an average node degree of 2.37, a network diameter of 11, and a clustering coefficient of 0.185. Following PPI network construction, the MCODE algorithm was applied in Cytoscape to identify network modules and hub genes within the connected component (MCODE score = 2.800). MCODE analysis highlighted a cluster of highly interconnected genes (hub genes), including *DVL1*, *PRKCZ*, *TAS1R3*, *GABRD*, *CFAP74*, and *CPTP* (Figure 2). These hub genes may play central roles in the functional network of the duplicated region. According to the Scout gene database, the duplicated region contains 19 OMIM morbid genes, including 9 with autosomal recessive (AR) and 10 with autosomal dominant (AD) inheritance patterns. Among the hub genes identified in the PPI network (*CFAP74, GABRD, PRKCZ, DVL1, TAS1R3, CPTP*), only *CFAP74*, *GABRD*, and *DVL1* are classified as OMIM morbid genes. Of these, *GABRD* and *DVL1* are exclusively associated with autosomal dominant inheritance patterns, further supporting their relevance as candidate genes in the context of heterozygous duplications.

#### 3.3.2 Exploring CNV Using Decipher

We systematically reviewed all reported duplication CNVs overlapping this region. Based on DECIPHER data (hg38), a total of 373 copy number and sequence variations—including 59 SNV/indels (less than 50 bp), 218 deletions, and 89 duplications—have been reported within this region. Among the duplications, 20 CNVs are classified as pathogenic or likely pathogenic, and 34 as variants of uncertain significance (VUS). The smallest pathogenic or likely pathogenic CNV reported in this region is 68.05 kb, while the largest reaches 7.94 Mb. Notably, three pathogenic CNVs are less than 1 Mb in size and include the morbid genes *PANK4, PEX10, PRDM16, SKI, ATAD3A, CFAP74, GABRD, GNB1, TMEM240*, and *VWA1*. The smallest pathogenic duplication encompasses three genes—*ATAD3A, ATAD3B*, and *ATAD3C*—with *ATAD3A* recognised as a morbid gene in OMIM. Table 1 provides a comparative summary of phenotypes associated with each duplication CNV.

**TABLE 1:** Pathogenic and likely pathogenic duplication CNVs in the studied region (DECIPHER database).

DECIPHER patient	Sex	Location	Size	Inheritance/genotype	Pathogenicity/contribution	Phenotype(s)
264,192	46XX	1:875,661–6,076,860	5.20 Mb	Unknown	Pathogenic	Generalised‐onset seizure, Global developmental delay, Moderately short stature, Poor speech
		GRCh38		Heterozygous	Partial	
		Duplication				
270,940	46XY	1:1,456,616–1,524,663	68.05 kb	De novo (parentage confirmed)	Pathogenic	Cardiomegaly, Congenital contracture, Generalised opacification of the cornea, Hypertonia, Hypertrophic cardiomyopathy, Hypospadias, Muscle abnormality related to mitochondrial dysfunction, Overlapping toe, Thoracic scoliosis, Toe clinodactyly
		GRCh38		Heterozygous	Full	
		Duplication				
278,905	46XY	1:917,483–5,518,089	4.60 Mb	Maternally inherited	Pathogenic	Intellectual disability, moderate
		GRCh38		Heterozygous		
		Duplication				
331,385	46XY	1:2,112,225–3,227,319	1.12 Mb	Maternally inherited	Likely pathogenic	Mild global developmental delay, obsolete Impaired social interactions
		GRCh38		Heterozygous		
		Duplication				
331,665	46XX	1:2,892,366–3,367,752	475.39 kb	De novo (unconfirmed parentage)	Pathogenic	Motor delay
		GRCh38		Heterozygous		
		Duplication				
331,697	46XY	1:629,046–2,349,810	1.72 Mb	Unknown	Likely pathogenic	
		GRCh38		Heterozygous		
		Duplication				
337,918	46XY	1:824,382–2,563,755	1.74 Mb	Unknown	Likely pathogenic	Feeding difficulties, Hypotelorism, Neonatal hypotonia, Retrognathia
		GRCh38		Heterozygous		
		Triplication				
394,315	46XY	1:10,001–6,905,674	6.90 Mb	Imbalance arising from a balanced parental rearrangement	Likely pathogenic	Abnormal erythrocyte morphology, Broad forehead, Depressed nasal bridge, Hypertelorism, Intellectual disability, Preauricular pit, Ptosis, Small forehead, Strabismus
		GRCh38		Heterozygous		
		Duplication				
395,321	46XX	1:10,001–6,905,674	6.90 Mb	Imbalance arising from a balanced parental rearrangement	Likely pathogenic	Abnormal erythrocyte morphology, Brachydactyly, Hypertelorism, Recurrent infections, Sacral dimple, Strabismus
		GRCh38		Heterozygous		
		Duplication				
395,323	46XY	1:10,001–6,905,674	6.90 Mb	Imbalance arising from a balanced parental rearrangement	Likely pathogenic	Abnormal erythrocyte morphology, Anteverted nares, Broad forehead, Cryptorchidism, Depressed nasal bridge, Hypertelorism, Intellectual disability, Preauricular pit, Ptosis, Small forehead, Strabismus
		GRCh38		Heterozygous		
		Duplication				
398,345	46XX	1:10,001–6,905,674	6.90 Mb	Unknown	Likely pathogenic	2–3 toe syndactyly, Atrial septal defect, Craniosynostosis, Delayed speech and language development, Intellectual disability, Microcephaly, Narrow forehead, Prominent metopic ridge, Proportionate short stature, Short palpebral fissure, Trigonocephaly
		GRCh38		Heterozygous		
		Duplication				
400,880	46XX	1:871,284–8,812,156	7.94 Mb	Unknown	Likely pathogenic	Abnormal pinna morphology, Brachycephaly, Constipation, Deeply set eye, Delayed closure of the anterior fontanelle, Delayed speech and language development, EEG abnormality, Flat occiput, Frontal bossing, Hypotonia, Intellectual disability, Microcephaly, Midface retrusion, Proportionate short stature, Recurrent infections, Short palpebral fissure, Synophrys, Underdeveloped nasal alae, Wide nasal bridge
		GRCh38		Heterozygous		
		Duplication				
438,664	46XY	1:3,077,175–5,030,655	1.95 Mb	Unknown	Likely pathogenic	
		GRCh38		Heterozygous		
		Duplication				
472,385	46XY	1:82,154–6,124,132	6.04 Mb	Unknown	Pathogenic	
		GRCh38		Heterozygous		
		Duplication				
472,620	46XY	1:863,579–5,739,571	4.88 Mb	Unknown	Pathogenic	
		GRCh38		Heterozygous		
		Duplication				
486,797	46XY	1:1,354,381–2,542,766	1.19 Mb	Paternally inherited	Likely pathogenic	Attention deficit hyperactivity disorder, Delayed puberty, Moderate global developmental delay, Seizure
		GRCh38		Heterozygous	Uncertain	
		Duplication				
501,184	46XX	1:787,938–2,400,650	1.61 Mb	De novo (parentage confirmed)	Pathogenic	
		GRCh38		Heterozygous		
		Amplification				
501,880	46XY	1:2,235,714–3,221,916	986.20 kb	Unknown	Likely pathogenic	Specific learning disability
		GRCh38		Heterozygous		
		Duplication				
503,091	46XX	1:754,622–3,193,436	2.44 Mb	De novo (parentage confirmed)	Likely pathogenic	
		GRCh38		Heterozygous	Partial	
		Duplication				
549,911	46XY	1:866,156–3,521,941	2.66 Mb	Unknown	Likely pathogenic	
		GRCh38		Heterozygous		
		Duplication				

#### 3.3.3 Prioritisation of Candidate Genes and Triplosensitivity Analysis

**FIGURE 3:**
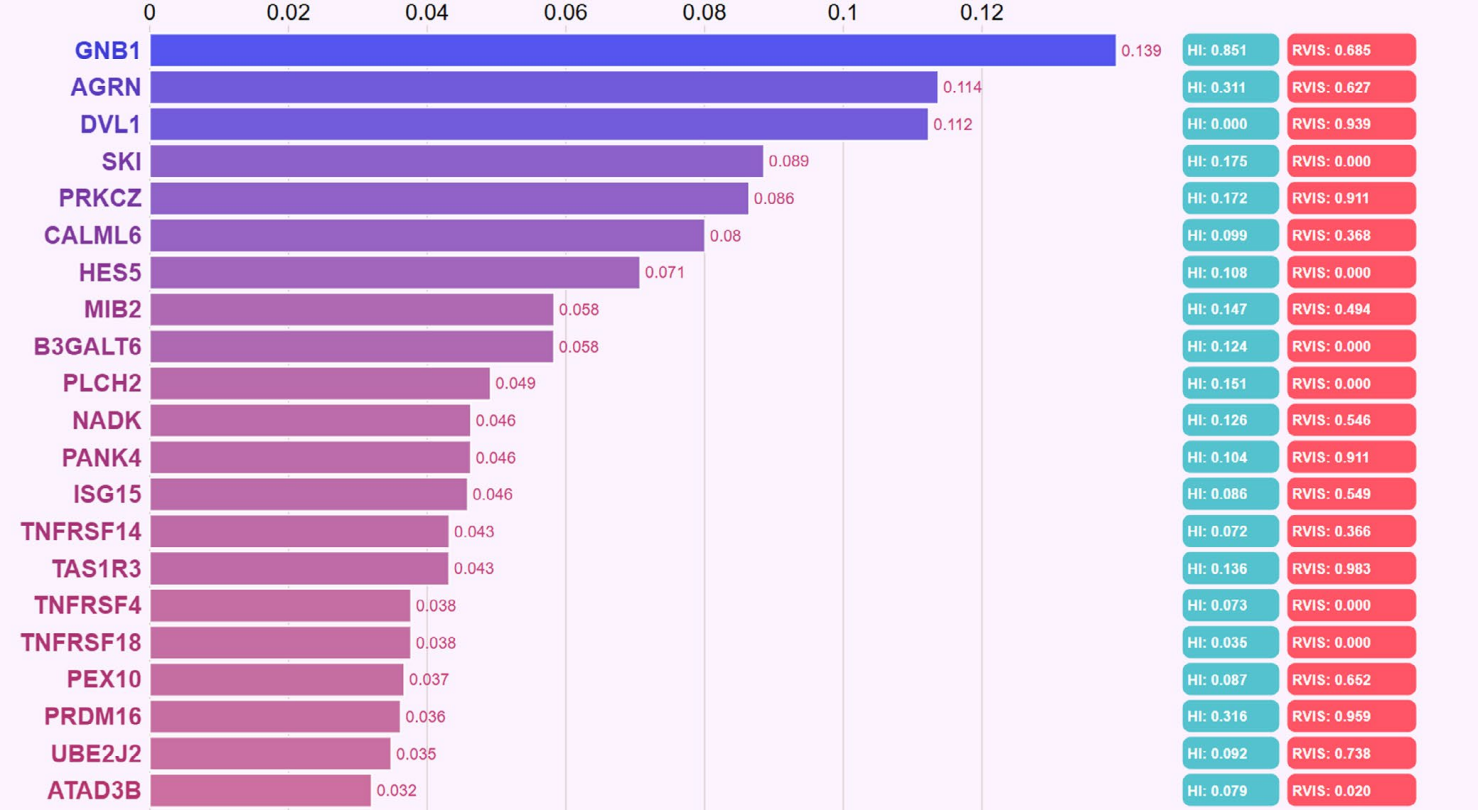
Phenolyzer‐based prioritisation of genes within the duplicated region using 13 patient‐specific phenotypes (HPO terms). Bars represent the Phenolyzer prioritisation scores for each gene. GNB1 received the highest score among all 89 candidate genes, suggesting its strong association with the observed clinical features. HI, Haploinsufficiency score; RVIS, Residual Variation Intolerance Score.

Using Phenolyzer, we analysed 13 patient phenotypes together with the 89 genes located in the duplicated region to identify the genes most strongly associated with the observed clinical features. This analysis prioritised GNB1 as the top candidate gene (Figure 3). Furthermore, triplosensitivity assessment in the DECIPHER database revealed that among all genes within the duplicated interval, GNB1 was the only gene with a high and significant triplosensitivity score (pTriplo = 1.00). None of the other genes in this region showed a comparable level of predicted triplosensitivity, highlighting GNB1 as the principal dosage‐sensitive gene and supporting its potential relevance to the observed phenotype in our patient.

#### 3.3.4 Pathway Enrichment Analysis

To further investigate the biological relevance of the duplicated region, we performed pathway enrichment analysis using Enrichr. The list of 89 genes identified within the 1p36 duplicated region (based on Ensembl, hg38) was submitted to the Enrichr platform. Using the WikiPathways 2024 Human database, significant enrichment was observed for the ‘1P36 Copy Number Variation Syndrome’ pathway (WP5345), with an adjusted *p*‐value of 6.420 × 10 ^−111^. Interestingly, the ‘1P36 Copy Number Variation Syndrome’ pathway (WP5345), which is primarily defined based on studies of 1p36 deletions, was also significantly enriched in our gene list. This indicates that the genes duplicated in our case overlap with those implicated in the well‐characterised deletion syndrome; however, the biological and clinical consequences of duplication may differ from those of deletion, highlighting the need for further investigation into dosage effects within this region.

### 3.4 Use of AI‐Assisted Tools in Writing

We used ChatGPT for language editing and clarity improvements in early drafts of the manuscript. All AI‐generated suggestions were reviewed, verified, and edited by the authors, who take full responsibility for the content. No AI tools were used for study design, data collection, statistical analysis, figure/table generation, or drawing scientific conclusions, and no patient‐identifiable or confidential data were entered into the tool.

## 4 Discussion

In this report, according to the best of our knowledge, we present the first Iranian case of pathogenic gain of 2.3 Mb resulting in developmental delay and dysmorphic features. This anomaly was identified using array CGH and validated through quantitative Real‐Time PCR, marking the first such case as reported in the DECIPHER database. The child displayed distinctive physical characteristics, encompassing micrognathia, pronounced lower lip, bulbous nose, ample cheeks, widely spaced teeth, unilateral club foot, and esotropia. Additionally, left ventriculomegaly, as observed in the MRI images, was noted as a non‐specific brain feature. Genotype–phenotype correlation in 1p36 CNVs is further complicated by phenotypic heterogeneity. In addition to non‐specific findings such as developmental delay and microcephaly, duplications in this region have been associated with unique features including craniosynostosis, epilepsy, and various dysmorphisms [19, 20, 21, 22]. Moreover, similar clinical manifestations have been reported in cases with deletions, duplications, and even tetrasomy of 1p36 in the DECIPHER database, reflecting the locus's high functional complexity. Therefore, we identified several candidate genes including *GABRD, DVL1*, and *GNB1* through integrative bioinformatic analyses that are likely to contribute to the observed clinical phenotype. *GABRD* encodes the delta (δ) subunit of the gamma‐aminobutyric acid (GABA) type A receptor, a major inhibitory neurotransmitter receptor in the central nervous system (CNS) (NCBI Gene ID: 2563) [23]. Heterozygous mutations in *GABRD* have been linked to a range of neurological and psychiatric disorders, most notably Generalised Epilepsy with Febrile Seizures Plus (GEFS+, OMIM #613060). Recent studies have challenged the traditional view that increased GABAergic inhibition is universally protective against seizures. δ‐Containing GABA‐A receptors, encoded by *GABRD*, mediate tonic inhibition, and gain‐of‐function variants in *GABRD* have been shown to paradoxically promote absence seizures, both in human patients and animal models [24]. These variants lead to increased tonic GABAergic currents in thalamocortical neurons, altering network excitability and facilitating seizure generation. Similar clinical and electrographic features are observed in patients with loss‐of‐function *SLC6A1* variants, which also elevate ambient GABA and enhance δ‐GABA‐A receptor activation [24]. Given that gain‐of‐function and increased dosage of *GABRD* can lead to enhanced tonic inhibition and seizure susceptibility, it is plausible that the duplication of this gene in our patient may underlie some of the observed clinical features, including epilepsy.

*DVL1* belongs to a family of highly conserved proteins (*DVL1, DVL2, DVL3*) that are key mediators of the *Wnt* signalling pathway. These proteins act as intracellular scaffolds immediately downstream of *Wnt* receptors and determine the directionality of Wnt signalling into canonical and non‐canonical pathways. While functional redundancy exists among the paralogs, animal models show that *Dvl2* and *Dvl3* are critical for development, with loss leading to major congenital anomalies, whereas *Dvl1* loss has milder effects [25]. Heterozygous truncating mutations in *DVL1* cause autosomal dominant Robinow syndrome‐2 that is a skeletal dysplasia characterised by distinctive facial features, including midface hypoplasia, hypertelorism, a short nose, and a broad mouth, known collectively as fetal facies. Additional features include mesomelic dwarfism, macrocephaly, gingival hypertrophy, dental malocclusion, genital hypoplasia, and brachydactyly [25, 26, 27, 28, 29]. In vitro cellular expression studies demonstrated that co‐transfection of the mutant and wildtype *DVL1* resulted in increased activation of the canonical WNT signalling pathway, suggesting a gain‐of‐function effect [25]. These findings indicate that pathogenic *DVL1* variants can aberrantly activate WNT signalling even in the presence of a normal allele. Therefore, it is plausible that *DVL1* duplication may similarly lead to increased gene dosage and excessive WNT pathway activation, thereby contributing to the clinical phenotype observed in our patient. This underscores the critical importance of *DVL1* dosage and WNT pathway regulation in neurodevelopmental and dysmorphic features. *GNB1* encodes the beta subunit of heterotrimeric guanine nucleotide‐binding (G) proteins, which act as binary molecular switches in signal transduction [30]. Pathogenic variants in *GNB1* cause Intellectual Developmental Disorder, Autosomal Dominant‐42 (OMIM #616973), characterised by global developmental delay and intellectual impairment. Additional features may include hypotonia, seizures, strabismus, cortical visual impairment, and autistic traits. While previous studies have established that loss‐of‐function mutations in *GNB1* cause global developmental delay, epilepsy, and a range of neurodevelopmental phenotypes by disrupting G protein signalling [31], the clinical consequences of *GNB1* duplication or increased gene dosage remain largely unexplored. Therefore, our findings extend the current knowledge by suggesting that increased *GNB1* dosage may also contribute to neurodevelopmental disorders, possibly through a distinct mechanism involving enhanced G protein signalling or dosage imbalance. The observed phenotype in 1p36 duplications may result from the effect of a single gene or from the combined interaction of multiple genes within the duplicated region. For example, *GABRD* is absent in the smallest duplications and these patients do not exhibit seizures; however, seizures are observed in cases where larger duplications include *GABRD*, indicating a gene‐specific contribution. In addition to size‐ and content‐dependent effects, the genomic architecture and mechanistic consequences of a copy number variation (CNV) can be critical determinants of pathogenicity. Notably, chromosome 1p36.33 duplication syndrome (OMIM: 618815), involving the ATAD3 gene cluster, overlaps with the CNV interval identified in our patient. This locus lies within a segment of high sequence homology that is prone to non‐allelic homologous recombination (NAHR). That study described highly homologous interval within the ATAD3 region that can serve as the recombination junction, generating an ATAD3A–C fusion gene. Clinically, reported cases exhibit a severe neonatal multisystem phenotype characterised by cardiomyopathy, corneal opacities/clouding, hypotonia, seizures, white‐matter abnormalities (often accompanied by metabolic disturbances such as lactic acidosis), and early lethality [19]. In contrast, our proband's clinical presentation showed limited phenotypic concordance with the classic ATAD3‐cluster duplication phenotype described in the literature. Mechanistically, the same study proposes that the fulminant neonatal phenotype is driven predominantly by a dominant‐negative effect of the ATAD3A–C fusion protein generated by these recurrent NAHR‐mediated duplications, emphasising that duplication architecture (i.e., whether a pathogenic fusion is created) is critical for pathogenicity, with increased ATAD3B dosage alone unlikely to explain the severe neonatal disease.

Taken together, these observations suggest that CNV‐associated phenotypes are not determined by size alone. In some cases, a single key gene may act as the primary driver, with clinical features largely reflecting disruption or altered dosage of that gene. However, even relatively small CNVs can differ markedly in clinical impact depending on the mechanism of formation and the resulting pathogenic architecture—for example, whether the rearrangement is fusion‐forming and capable of a dominant‐negative effect, versus producing a more conventional dosage change. As CNV size increases, additional genes may be included, and phenotypic variability may emerge from gene–gene interactions and pathway‐level perturbations, where the combined effects of multiple dosage changes modulate the ultimate clinical outcome.

## 5 Conclusion

Our findings highlight the complexity of genotype–phenotype correlations in 1p36 duplications and underscore the importance of combining genomic data with clinical and computational tools for accurate interpretation of rare copy number variants. Further studies, including functional validation and larger cohorts, are warranted to better elucidate the pathogenic mechanisms and improve clinical management of patients with 1p36 duplications.

## Author Contributions

Conceptualization: Bita Barazandeh Shirvan, Javad Akhondian, Mehran Beiraghi Toosi, Majid Mojarrad. Methodology: Bita Barazandeh Shirvan. Software: Mir Salar Kahaei. Investigation: Atieh Eslahi, Masoome Alerasool, Javad Akhondian, Mobina Arabi, Farnoosh Ebrahimzadeh, Mehran Beiraghi Toosi. Validation: Narges Hashemi. Formal analysis: Mir Salar Kahaei. Visualisation: Mir Salar Kahaei. Resources: Atieh Eslahi, Majid Mojarrad. Supervision: Mehran Beiraghi Toosi, Majid Mojarrad. Writing – original draft: Bita Barazandeh Shirvan, Sepideh Tabarestani. Writing – review and editing: Razie Rezaie.

## Funding

The authors have nothing to report.

## Conflicts of Interest

The authors declare no conflicts of interest.

## Supporting information


**Table S1:** The sequence of primers used for Quantitative Real‐Time PCR.
**Table S2:** List of 13 clinical phenotypes (HPO terms) used as input for gene prioritisation in Phenolyzer analysis. The corresponding HPO identifiers are provided for each phenotype.
**Table S3:** ACMG/ClinGen scoring.

## Data Availability

The data that support the findings of this study are available from the corresponding author upon reasonable request.
