# Supplementary material for: Comprehensive Clinical, Diagnostic, and In Silico Assessment of a Novel 1p36.33p36.32 Copy Number Variant
Source: J Cell Mol Med. 2026 Feb 25;30(4):e71079. doi: 10.1111/jcmm.71079 (PMC12933876; doi:10.1111/jcmm.71079)
Supplement: Supplementary file 1 — Table S1: The sequence of primers used for Quantitative Real‐Time PCR. Table S2: List of 13 clinical phenotypes (HPO terms) used as input for gene prioritisation in Phenolyzer analysis. The corresponding HPO identifiers are provided for each phenotype. Table S3: ACMG/ClinGen scoring. [file JCMM-30-e71079-s001.docx]

| Primer name | Forward Sequence (5’-3’) | Reverse Sequence (5’-3’) | Size (bp) | Purpose |
| --- | --- | --- | --- | --- |
| ***PRKCZ*** | GACCTTAAGCCAGTTATCGA | CACCTACCTCGTCATCATG | 196 | Target gene |
| ***KMT2D*** | GGCCAAGGGTGATGATGAAC | TCCCGTTCAGCCTTCTCATT | 195 | Reference gene |

**Supplementary table 1.** The sequence of primers used for Quantitative Real-Time PCR.

**Supplementary table 2.** List of 13 clinical phenotypes (HPO terms) used as input for gene prioritization in Phenolyzer analysis. The corresponding HPO identifiers are provided for each phenotype.

| **Phenotype** | **HPO** |
| --- | --- |
| Broad Thumb | HP:0011304 |
| Low-set ears | HP:0000369 |
| micrognathia | HP:0000347 |
| Everted lower lip vermilion | HP:0000232 |
| Bulbous nose | HP:0000414 |
| Full cheeks | HP:0000293 |
| Widely spaced teeth | HP:0000687 |
| Talipes equinovarus | HP:0001762 |
| Esotropia | HP:0000565 |
| No social interaction | HP:0008763 |
| Delayed speech and language development | HP:0000750 |
| Seizure | HP:0001250 |
| Ventriculomegaly | HP:0002119 |

**Supplementary table 3.** ACMG/ClinGen scoring

| **Criterion Code** | **Description** | **Score** | **Key Findings** |
| --- | --- | --- | --- |
| **1A** | Contains protein-coding or other known functionally important elements | 0.00 | Overlaps with 63 protein-coding genes, and 2 ClinGen dosage-sensitive regions |
| **1B** | Does NOT contain protein-coding or known functional elements | 0.00 | Unmet |
| **2A** | Complete overlap with established genomic region | 0.00 | Unmet |
| **2B** | Partial overlap with TS region without causative gene | 0.00 | The following Triplosensitive regions or genes partially overlap with the variants:  Region id: ISCA-37434, Region name: 1p36 terminal region (includes GABRD), Triplosensitivity score: 2. |
| **2H** | Haploinsufficient gene fully contained in gain | 0.00 | 15 HI genes (*SKI*, *PERM1*, *GNB1*, etc.) |
| **2K** | Breakpoint within HI gene, phenotype consistent | 0.00 | Breakpoint in *PRDM16*, phenotype not matching LOF |
| **2L** | Breakpoint in gene without clinical significance | 0.00 | *PRDM16* |
| **3C** | ≥50 protein-coding genes included | +0.90 | Total: 59 genes (not from same family) |
| **3A** | >35 and <50 genes | — | Unmet |
| **3B** | >25 and <35 genes | — | Unmet |
| **4L** | Case–control enrichment | +0.15 | Significant enrichment among affected individuals |
| **4O** | Overlap with common population variants | 0.00 | No variants in gnomAD/DGV; allele frequency = 0 |
| **4M, 4N** | Population overlap subrules | — | Unmet |
